# Microbial Transformations of 7-Hydroxyflavanone

**DOI:** 10.1100/2012/254929

**Published:** 2012-04-30

**Authors:** Edyta Kostrzewa-Susłow, Tomasz Janeczko

**Affiliations:** Department of Chemistry, Wrocław University of Environmental and Life Sciences, Norwida 25, 50-375 Wrocław, Poland

## Abstract

Microbial transformations of racemic 7-hydroxyflavanone using strains of genus *Aspergillus (A. niger KB, A. niger 13/5, A. ochraceus 456)* and the species *Penicillium chermesinum 113* were studied. The products of *O*-methylation, *O*-methylation along with hydroxylation at C-3′ and C-4′, reduction of the carbonyl group, reduction of the carbonyl group along with hydroxylation at C-5, and dehydrogenation of C-2 and C-3 were obtained. Most of the products (with the exception of the *O*-methylation one) have stronger antioxidant properties than the initial substrate.

## 1. Introduction

Flavonoids comprise a large group of secondary metabolites derived from phenylalanine. They are commonly found in plants, where their biogenesis takes place under normal physiological conditions (e.g., synthesis of pigments in flowers), but also may be a part of environmental stress response (e.g., synthesis of phytoalexins) [1–3]. Although the presence of natural flavonoids in animals and people has not been confirmed so far, their multidirectional influence on many important processes in these organisms was proved and reported by several research institutes all over the world [4–7]. Among many review articles about flavonoids, the following two: “*The Flavonoids*” [8] and “*The Handbook of Natural Flavonoids*” [1], are particularly rich in information. They present a couple of thousands of known compounds of this group, including their structures, separation methods, and identification details. On the basis of many cited papers the authors describe chemical and biological properties of flavonoids, including their influence on capillary vessels structure, on functioning of heart, liver, and kidneys. They also report diuretic, antibacterial, antiviral, immunomodulatory, anticancer, hypoglycemic, and antioxidant properties of flavonoids.

Practical application of flavonoids in pharmacy or in medicine is often considerably limited due to their low solubility, inefficient transport across biological membranes or low stability. Biotransformation of flavonoid compounds may be a natural method of modification of their structures, aiming at greater structural diversity and better bioaccessibility. It also gives a possibility to trace metabolic transformation of flavonoids [9–12].

Enzymatic systems of microorganisms are capable of different reactions, including hydrolysis, carbonyl group reduction, hydroxylation, *O*-methylation, oxidation, isomerization, and also formation or breakage of C–C and C–heteroatom bonds [13, 14].

Ibrahim and Abul-Hajj described biotransformation of monohydroxyflavones with substituents in the A-ring using the strain of *Streptomyces fulvissimus *(NRRL 1453B). They obtained products of hydroxylation at 4′ or both 3′ and 4′ positions in the B-ring. They have also observed that the distance between the carbonyl and hydroxyl groups in the A-ring has impact on the reaction speed and efficiency [15]. Herath and coworkers transformed 7-hydroxyflavone into 7,4′-dihydroxyflavone by means of the strain of *A. alliaceus* (ATCC 10060), whereas, using the strain of *Beauveria bassiana* (ATCC 7159) led to formation of 7-*O*-*β*-D-4-*O*-Methylglucopyranoside and 4′-hydroxyflavone 7-*O*-*β*-D-4-*O*-methylglucopyranoside. *O*-methylation of 7-hydroxyflavone was catalyzed by *Nocardia species* (NRRL 5646). The other substrate—3-hydroxyflavone—was transformed by means of *B. bassiana* (ATCC 13144) into 3,4′-dihydroxyflavone and flavone 3-*O*-*β*-D-4-*O*-methylglucopyranoside [16]. The same strain metabolized 5-hydroxyflavone into 5,4′-dihydroxyflavone and 4′-hydroxyflavone 5-*O*-*β*-D-4-*O*-methylglucopyranoside, whereas 6-hydroxyflavone was transformed into 6-hydroxyflavanone, flavone 3-*O*-*β*-D-4-*O*-methylglucopyranoside, and racemic flavanone 6-*O*-*β*-D-4-*O*-methylglucopyranoside [17].

The substrates that possess several hydroxyl groups in the flavonoid skeleton, like quercetin or fisetin, in the culture of *S. griseus* (ATCC 13273) underwent regioselective either hydroxylation or methylation [18]. In the other paper regioselective 4′-*O*-demethylation of tangeretin and 3-hydroxytangeretin catalyzed by *A. niger* was reported [19].

In this paper we present the study in which we used the filamentous fungi of genera *Aspergillus* and *Penicillium* to enzymatic transformation of a xenobiotic substrate (7-hydroxyflavanone). The aim of this research was to obtain new, not known in the literature, compounds with stronger antioxidant activity than the initial substrate, to establish the metabolic pathways of 7-hydroxyflavanone in filamentous fungi cultures and also to determine the reactions specific for the tested microorganisms.

## 2. Materials and Methods

### 2.1. Analysis

The course of microbial transformation was monitored by TLC (SiO_2_, DC Alufolien Kieselgel 60 F_254_, Merck, Darmstadt, Germany). Chromatograms were developed using the following developing systems: hexane : ethyl acetate (7 : 3), dichloromethane : ethyl acetate (1 : 1), toluene : diethyl ether (4 : 1). Column chromatography (SiO_2_, Kieselgel 60, 230–400 mesh, 40–63 *μ*m, Merck) was performed using the same eluents. ^1^H NMR and ^13^C NMR spectra were recorded with a Bruker Avance DRX 300 spectrometer. IR spectra were determined with a Mattson IR 300 Thermo Nicolet spectrometer. Mass spectra were obtained using high-resolution electrospray ionization (ESI^+^-MS) (Waters LCT Premier XE mass spectrometer).

 HPLC analyses were performed with a Waters 2690 instrument equipped with Waters 996 photodiode array detector, using ODS 2 column (4.6 × 250 mm, Waters) and a Guard-Pak Inserts *μ*Bondapak C18 pre-column. Separation conditions were as follows: gradient elution, using 80% of acetonitrile in 4.5% formic acid solution (eluent A) and 4.5% formic acid (eluent B); flow, 1 mL/min; detection wavelength 280 nm; program: 0–7 min, 10% A 90% B; 7–10 min, 50% A 50% B; 10–13 min, 60% A 40% B; 13–15 min, 70% A 30% B; 15–20 min 80% A 20% B; 20–30 min 90% A 10% B; 30–40 min, 100% A. Melting points were determined with a Boetius apparatus (Kofler block). Antioxidant properties were measured with a Cintra 20 spectrometer (GBC, Melbourne, Australia).

### 2.2. Materials

The racemic substrate for biotransformation—7-hydroxyflavanone **(1)**—was purchased from Aldrich.

#### 2.2.1. 7-Hydroxyflavanone **(1)**

C_15_H_12_O_3_; melting point 188–190°C; ^1^H NMR see Table 1; ^13^C NMR see Table 2, IR (KBr, *ν*_max⁡_, cm^−1^): 3413 (O–H, stretch), 1651 (C=O, stretch), 1577 (C–C, stretch, aromatic), 1260 (–C–OH, bending), and 1062 (–C–OH, stretch); HRESI-MS [M + H^+^] (calculated/found) (*m/z* 241.0865/241.0860).

#### 2.2.2. Microorganisms

For our research we used a wild strain of *A. niger KB* and a UV mutant of *A. niger* (13/5). The *KB* strain was obtained from the collection of the Department of Biotechnology and Food Microbiology of Wrocław University of Environmental and Life Sciences (Poland) and the strain *13/5* from the University of Life Sciences in Lublin (Poland). The microorganisms were maintained on potato slants (sterilized piece of potato) at 5°C.

The wild strains *A. ochraceus 456* and *P. chermesinum 113* were obtained from the collection of the Department of Chemistry of Wrocław University of Environmental and Life Sciences (Poland). The microorganisms were maintained on agar slants at 5°C.

## 3. Biotransformations

### 3.1. Screening Procedure

 Cultivation media consisted of 3% glucose (The Industrial and Trading Enterprise “Stanlab” Co. Ltd., Poland) and 1% peptobac (BTL sp. z o.o., Poland) in water. The microorganisms were transferred from the slants to 500 mL Erlenmayer flasks, each containing 200 mL of the medium. Preincubation was performed at 25°C for 24–48 h. Then portions of 1 mL of the culture solution were transferred to inoculate 500 mL flasks, each containing 200 mL of the medium. After cultivation at 25°C for 24 hours on a rotary shaker, 10 mg of a substrate, dissolved in 0.5 mL of THF, was added to the grown culture. Control cultivation with no substrate was also performed. After 3, 6 and 9 days of incubation under the above conditions, portions of 5 mL of the transformation mixture were withdrawn and extracted with ethyl acetate (3 × 3 mL). The extracts were dried over MgSO_4_ (5 min), concentrated in vacuo and analyzed by TLC. Quantitative analyses of the mixtures were performed by means of HPLC. Calibration curves for quantitative analyses were prepared using isolated and purified biotransformation products as standards.

### 3.2. Preparative Biotransformation

 Portions of 1 mL of the Preincubation culture solution were used to inoculate three 2000 mL flasks, each containing 500 mL of the cultivation medium. The cultures were incubated at 25°C for 48 hours on a rotary shaker. Then 50 mg of the substrate dissolved in 2.5 mL of THF was added to each flask (100 mg of the substrate per 1 L of the cultivation mixture). After 3, 6, or 9 days of incubation the mixtures were extracted with ethyl acetate (3 × 200 mL), dried (MgSO_4_), and concentrated in vacuo. The transformation products were separated by column chromatography. Pure products were identified by means of spectral analyses (TLC, ^1^H NMR, ^13^C NMR, IR).

Physical and spectral data of the products obtained are presented next.

#### 3.2.1. 7-Methoxyflavanone **(2)**

C_16_H_14_O_3_; melting point 89-90°C; 24% yield; purity 98% (HPLC); [*α*]_546_^20^ = 0, (*c* = 0.95, CH_3_OH); ^1^H NMR see Table 1; ^13^C NMR see Table 2; IR (KBr, *ν*_max⁡_, cm^−1^): 3032 (C–H stretch, aromatic), 1683 (C=O, stretch), 1614 (C–C, stretch, aromatic), and 1438 (C–C, stretch, aromatic); HRESI-MS [M + H^+^] (calculated/found) (*m/z* 255.1021/255.1009).

#### 3.2.2. 3′,4′-Dihydroxy-7-methoxyflavanone **(3)**

C_16_H_14_O_5_; oily liquid; 19% yield; purity 98% (HPLC); [*α*]_546_^20^ = 0, (*c* = 1.0, CH_3_OH); ^1^H NMR see Table 1; ^13^C NMR see Table 2; IR (KBr, *ν*_max⁡_, cm^−1^): 3420 (O–H, stretch), 1710 (C=O, stretch), 1569 (C–C, stretch, aromatic), 1316 (C–O, stretch), and 1020 (C–O–C, stretch); HRESI-MS [M + H^+^] (calculated/found) (*m/z* 287.1022/287.1016).

#### 3.2.3. 2,4-cis-7-Hydroxyflavan-4-ol **(4)**

C_15_H_14_O_3_; oily liquid; 74% yield; purity 98% (HPLC); [*α*]_546_^23^ = 0, (*c* = 0.98, CH_3_OH), ^1^H NMR see Table 1; ^13^C NMR see Table 2; IR (CH_2_Cl_2_, *ν*_max⁡_, cm^−1^): 3220 (O–H, stretch), 1621 (C–C, stretch, aromatic), and 1598 (C–C, stretch, aromatic); HRESI-MS [M + H^+^] (calculated/found) (*m/z* 243.1050/243.1043).

#### 3.2.4. 2,4-trans-5,7-dihydroxyflavan-4-ol **(5)**

 C_15_H_14_O_4_; melting point 275–277°C; 12% yield; purity 98% (HPLC); [*α*]_546_^20^ = 0, (*c* = 1.95, CH_3_OH); ^1^H NMR see Table 1; ^13^C NMR see Table 2; IR (KBr, *ν*_max⁡_, cm^−1^): 3460 (O–H, stretch), 1615 (C–C, stretch, aromatic), and 1590 (C–C, stretch, aromatic); HRESI-MS [M + H^+^] (calculated/found) (*m/z* 259.0923/259.0918).

#### 3.2.5. 7-Hydroxyflavone **(6)**

C_15_H_10_O_3_; melting point 246–248°C; 98% yield; purity 99% (HPLC); ^1^H NMR see Table 1; ^13^C NMR see Table 2; IR (KBr, *ν*_max⁡_, cm^−1^): 3412 (O–H, stretch), 2926 (O–H, stretch, intramolecular hydrogen bond), 1627 (C=O, stretch), 1454 (C–C, stretch, aromatic), and 773 (C–H, bending, aromatic); HRESI-MS [M + H^+^] (calculated/found) (*m/z* 239.0760/239.0755).

### 3.3. Measurement of Antioxidant Properties of the Substrate and the Products

A methanolic solution of DPPH (1,1-diphenyl-2-picryl-hydrazyl) of absorbance of about 1.00 was mixed with a proper amount of a tested flavonoid. After 20 min, disappearance of absorbance at 520 nm was measured. The initial concentration of DPPH was determined by means of calibration curve. The IC_50_ value (antiradical activity) was determined on the basis of graphs—DPPH radical reduction (expressed in %) as a function of concentration of the tested compound. IC_50_ means concentration of the antioxidant that reduces the initial concentration of DPPH by half.

## 4. Results and Discussion

At first screening tests on racemic 7-hydroxyflavanone **(1)** were performed, in which for catalysis we used enzymatic systems of twenty-seven microorganisms of *Aspergillus, Penicillium, Coryneum, Nectria, Chaetomium, Absidia, Spicaria,* and *Cryptosporiopsis* genera. The tests led to selection of four microorganisms capable of biotransformation of this substrate. The following microorganisms were selected: three strains of *Aspergillus* (a wild strain of *A. niger KB*, its UV-mutant-*A. niger 13/5*, and *A. ochraceus 456*) and the strain of *Penicillium chermesinum 113*.

**Scheme 1:**
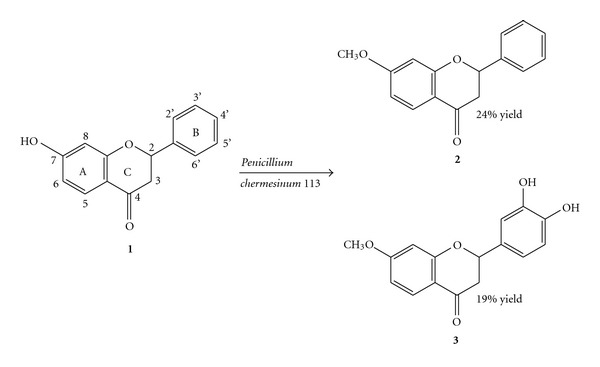


A six-day transformation of 7-hydroxyflavanone **(1)**, catalyzed by the strain of *P. chermesinum 113,* gave two products: 7-methoxyflavanone **(2)** in 24% yield and 3′,4′-dihydroxy-7-methoxyflavanone **(3)** in 19% yield (Scheme 1), with 8% of unreacted substrate **1 **remained. Both the biotransformation products and the unreacted substrate were racemic.

 The structures of newly formed products: 7-methoxyflavanone (**2**) and 3′,4′-dihydroxy-7-methoxyflavanone (**3**), were determined by means of ^1^H NMR, ^13^C NMR, and IR. In the IR spectrum of product **2** there is no absorption band at 3413 cm^−1^, observed for the substrate (**1**), which means that there is no hydroxyl group in product **2**. In the ^1^H NMR of **2** a new singlet at *δ* = 3.86 ppm appeared, integrating for 3 protons, which indicates the presence of a methoxyl group. The chemical shifts of A-ring protons did not considerably differ in the spectra of **1** and **2**, which unequivocally suggest that *O*-methylation occurs at the C-7 position. The presence of the methoxyl group was also confirmed by ^13^C NMR of **2**, where the signal at *δ* = 55.7 ppm is visible.

In the ^1^H NMR of 3′,4′-dihydroxy-7-methoxyflavanone (**3**) there are no considerable changes in the chemical shifts of H-5, H-6, and H-8 compared to the substrate (**1**); however, the presence of a methoxyl group at C-7 was confirmed by the singlet at *δ* = 3.84 ppm, integrating for three protons. Lack of H-3′ and H-4′ signals in the ^1^H NMR of **3 **and the shift of C-3′ signal (from *δ* = 129.7 ppm to *δ* = 135.2 ppm) and C-4′ signal (from *δ* = 129.6 ppm to *δ* = 135.4 ppm) in the ^13^C NMR of **3 **compared to the substrate confirms locations of the two hydroxyl groups at the C-3′ and C-4′ in the B-ring of product **3**.

**Scheme 2:**
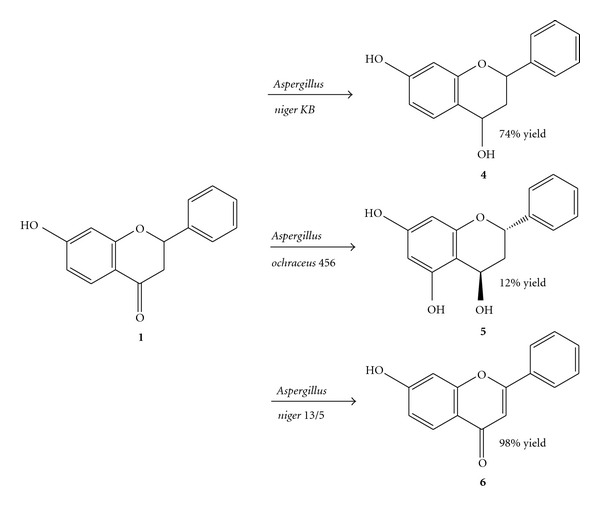


 Microbial transformation of 7-hydroxyflavanone (**1**) by means of *Aspergillus niger KB* led to reduction of the carbonyl group. After 9 days of biotransformation 7-hydroxyflavan-4-ol (**4**) was isolated in 74% yield (Scheme 2). The reaction was continued until all the substrate was consumed. In the ^1^H NMR spectrum of 7-hydroxyflavan-4-ol (**4**) there is a doublet of doublets at *δ* = 5.03 ppm observed, integrating for one proton and ascribed to H-4 (*J*_4,3ax_ = 9.9 Hz, *J*_4,3eq_ = 6.2 Hz). In the ^13^C NMR the signal of C-4 was shifted from *δ* = 193.1 ppm for 7-hydroxyflavanone (**1**) to *δ* = 65.6 ppm for 7-hydroxyflavan-4-ol (**4**). The chemical shifts of H-2, H-4, H-3_ax_, H-3_eq_ protons and their coupling constants in the ^1^H NMR spectrum of **4** were compared with the spectra of flavan-4-ol and 6-hydroxyflavan-4-ol (published data). These confirmed the structure of 2,4-*cis*-7-hydroxyflavan-4-ol (**4**) [20]. Flavan-4-ol and 6-hydroxyflavan-4-ol were obtained in biotransformation of flavanone and 6-hydroxyflavanone, respectively, catalyzed by *A. niger* KB. The structure of flavan-4-one was determined by means of X-ray analysis [20].

X-ray analysis of 2,4-*cis*-flavan-4-ol proved that the product was a racemic mixture of (2R, 4R) and (2S, 4S) isomers. The two crystallographically independent molecules have pseudoequatorial phenyl groups, whereas H-2 is in pseudoaxial position. This is confirmed by the values of the following angles: H*-2*–C-2–C-3–H3A = 67.11°; H-2–C-2–C-3–H3B = −174.21° and H2A–C2A–C3A–H3D = −67.80°; H2A–C2A–C3A–H3C = 173.04°, and also by the respective coupling constants: *J*_2-3eq_ = 1.83 Hz and *J*_2-3ax_ = 11.62 Hz (Table 3). In both molecules the 4-OH group is pseudo equatorial and 4-H is pseudo axial. This is confirmed by the respective coupling constants *J*_4-3eq_ = 6.27 Hz and *J*_4-3ax_ = 10.59 Hz (Table 3) as well as by the values of the angles: H-4–C-4–C-3–H3A = −46.61; H-4–C-4–C-3–H3B = −165.28 and H4A–C4A–C3A–H3D = 47.18; H4A–C4A–C3A–H3C = 166.34. (±)-7-Hydroxyflavan-4-ol (**4**) is an oily liquid; therefore it is not possible to carry out X-ray analysis of this compound. The 2,4-cis configuration of **4 **was ascribed by comparing the NMR chemical shifts and coupling constants of H-2, H-4, H-3_ax_, H-3_eq_ to the data for (±)-flavan-4-ol (Table 3).

The other product of biotransformation of 7-hydroxyflavanone (**1**) was found to be 5,7-dihydroxyflavan-4-ol (**5**). The reaction was catalyzed by the strain of *A. ochraceus 456* (Scheme 2). After 3 days of transformation the product of hydroxylation at C-5 along with the carbonyl group reduction (**5**) was isolated in 12% yield as a racemic mixture. The reaction was stopped after 3 days, because longer biotransformation time resulted in degradation of both the unreacted substrate and the newly formed product **5**. In the tenth day of the process there was no longer flavonoid structures observed in the reaction mixture.

 Hydroxylation at C-5 was confirmed in the ^1^H NMR spectrum of **5** by the signals at *δ* = 7.40 ppm and *δ* = 7.42 ppm (two one-proton doublets of doublets with *J* = 2.12 Hz) ascribed to H-6 and H-8, and also in the ^13^C NMR where the signal at *δ* = 129.9 ppm observed in the spectrum of substrate **1** was shifted to *δ* = 159.7 ppm in the spectrum of product **5**. In the ^1^H NMR of 5,7-dihydroxyflavan-4-ol (**5**) the presence of a one-proton doublet of doublets at *δ* = 5.52 ppm of H-4 indicates that the carbonyl group was reduced. This structural change was confirmed by the shift of the C-4 signal in the ^13^C NMR from *δ* = 193.1 ppm for the substrate to *δ* = 59.5 ppm for product **5**. Comparison of coupling constants of 2,4-*cis*-7-hydroxyflavan-4-ol (**4**) and product **5** (Table 3) led us to the structure of 2,4-*trans*-5,7-dihydroxyflavan-4-ol, with both phenyl and hydroxyl groups in pseudoequatorial positions.

 Transformation of 7-hydroxyflavanone (**1**) by means of *A. niger 13/5* led to the product of C-2 and C-3 dehydrogenation (**6**) (Scheme 2). 7-Hydroxyflavone (**6**) after 9 days of biotransformation was isolated in 98% yield. The substrate was fully consumed.

 In the ^1^H NMR of substrate **1** the signal of H-2 appears at *δ* = 5.43 ppm as a one-proton doublet of doublets with the coupling constants of *J* = 12.9 Hz and *J* = 3.0 Hz, whereas the signal of H-3_ax_ is observed at *δ* = 2.96 ppm as a doublet of doublets integrating for one proton with *J* = 16.9 Hz and *J* = 12.8 Hz, and finally the signal of H-3_eq_ is also a doublet of doublets at *δ* = 2.69 ppm with *J* = 16.9 Hz and *J* = 3.1 Hz. In the ^1^H NMR of the biotransformation product—7-hydroxyflavone (**6**)—there is no signal of H-2 and a new singlet of one proton appears at *δ* = 6.86 ppm, which is ascribed to the H-3. In the ^13^C NMR the signals of C-3 and C-2 are shifted from *δ* = 45.2 ppm and *δ* = 81.1 ppm for the substrate **1** to *δ* = 106.6 ppm (C-3) and *δ* = 162.8 ppm (C-2) for 7-hydroxyflavone (**6**). These changes unequivocally indicate the presence of a double bond between C-2 and C-3 in the C-ring of product **6**.

Comparison of UV spectra of 7-hydroxyflavanone (**1**) and its biotransformation products shows that the most significant shift of the maximum absorption bands to the longer wavelengths occurs for 7-hydroxyflavone (**6**) (Table 4), where conjugation of two chromophores: the carbonyl group and the C=C double bond, results in delocalization of *π* and *n* electrons, which facilitate electron excitations associated with *n*→ π* and *π*→ π* transitions.

For product **3** we observed an increase in absorption coefficient value compared to substrate **1** and a small bathochromic shift. In the case of the reduction products **4** and **5** a bathochromic shift of about Δ*λ*_max⁡_ = 8 nm (2nd band) is visible, which is slightly stronger for product **5**, with the additional hydroxyl group at C-5 (Table 4). We suppose that a hydrogen bond that is formed between the hydroxyl groups at C-5 and C-4 in compound **5** leads to formation of a specific “complex” with different location of the molecular energy levels than in the case when there is no specific interactions of this kind. A part of energy of the molecule is used for hydrogen bond formation. This results in lowering of both ground and excited states. The smaller difference in energy between these states is responsible for the red shift in the electronic spectra (bathochromic effect).

For substrate **1** and the obtained biotransformation products (**2–6**) the IC_50_ values were measured in order to establish and to compare their antioxidant properties (Table 5) [21]. The four products of microbial transformation of **1** showed higher antioxidant properties than the initial substrate. The highest antioxidant activity was observed for 3′,4′-dihydroxy-7-methoxyflavanone (**3**) (IC_50_ = 6.70). Such high antioxidant properties of **3** arise from the presence of two hydroxyl groups in the B-ring. The two hydroxyl groups in orthoposition in product **3** may facilitate chelating of metal ions [22–25].

## 5. Conclusions

(1) Microbial transformations of 7-hydroxyflavanone (**1**) as well as the earlier study on biotransformation of flavanone and 6-hydroxyflavanone using the strains of genus *Aspergillus* allowed to determine the ability of these microorganisms to perform specific reaction types:
  (i) the strain of *A. niger KB* catalyzes reduction of the carbonyl group;
  (ii) the strain of *A. niger 13/5* performs dehydrogenation of C-2 and C-3 positions of nonsubstituted flavanone and its monohydroxy derivatives.
(2) The strain *P. chermesinum 113,* apart from its typical reaction of hydroxylation in the B-ring, performed *O*-methylation of 7-hydroxyflavanone (**1**).
(3) A nontypical reaction of hydroxylation at C-5 along with carbonyl group reduction was observed for the strain *A. ochraceus 456*, which led to a new, not known in the literature, compound.
(4) The highest yields of products were observed for 7-hydroxyflavan-4-ol (**4**) (74% yield) and 7-hydroxyflavone (**6**) (98% yield). The reactions were catalyzed by the strains *A. niger KB *and* A. niger 13/5*, respectively.
(5) Microbial transformation of flavonoids may be used as a natural method leading to the derivatives of higher antioxidant properties than the initial substrates.

## Figures and Tables

**Table 1:** ^1^H NMR chemical shifts (*δ*) of compounds **1 ÷ 6**.

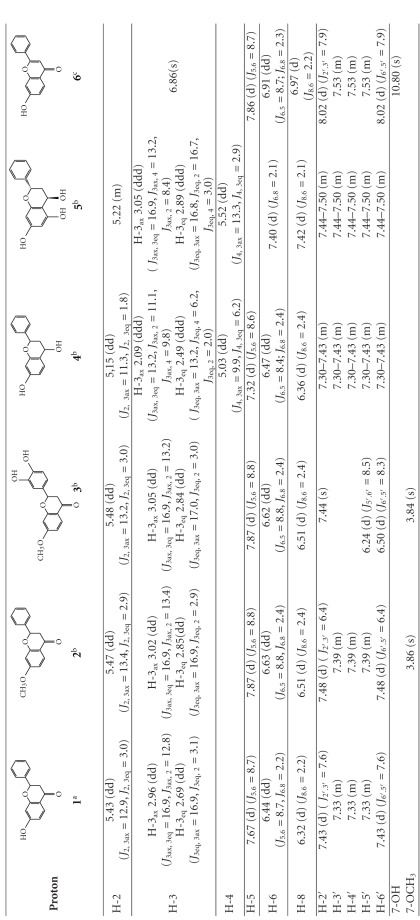

^a^Solvent CD_3_OD.

^b^Solvent CDCl_3_.

^c^Solvent DMSO-d_6_.

**Table 2:** ^13^C NMR chemical shifts (*δ*) of compounds **1 ÷ 6**.

Carbon	Compounds					
	1^a^	2^b^	3^b^	4^b^	5^b^	6^c^
C-2	81.1	80.2	80.0	77.0	73.8	162.8
C-3	45.2	44.3	44.2	40.3	37.8	106.6
C-4	193.1	192.2	190.8	65.6	59.5	176.4
C-5	129.9	129.7	128.8	128.3	159.7	126.5
C-6	111.9	110.1	110.3	108.8	92.0	115.1
C-7	166.9	168.0	166.2	156.3	161.5	161.9
C-8	103.9	100.9	100.9	103.0	93.5	102.6
C-9	165.4	162.2	163.6	155.4	156.5	157.5
C-10	115.1	126.2	114.7	118.7	106.3	116.1
C-1′	140.7	130.3	138.7	140.4	141.0	131.3
C-2′	127.4	127.9	115.5	126.0	126.3	126.2
C-3′	129.7	130.1	135.2	128.7	128.8	129.1
C-4′	129.6	128.8	135.4	128.3	128.1	131.6
C-5′	129.7	130.1	115.4	128.7	128.8	129.1
C-6′	127.4	127.9	115.9	126.0	126.3	126.2
OCH_3_		55.7	55.6			

^a^Solvent CD_3_OD.

^b^Solvent CDCl_3_.

^c^Solvent DMSO-d_6_.

**Table 3:** Selected ^1^H NMR data for 2,4-*cis*-7-hydroxyflavan-4-ol **(4)**, 2,4-*trans*-5,7-dihydroxyflavan-4-ol **(5)**, and 2,4-*cis*-flavan-4-ol.

	*δ* H-2	*δ* H-4	*δ* H-3_ax_	*δ* H-3_eq_	*J*_3ax-3eq_	*J*_2-3ax_	*J*_2-3eq_	*J*_4-3ax_	*J*_4-3eq_
2,4-*cis*-Flavan-4-ol	5.17	5.08	2.13	2.51	13.06	11.62	1.83	10.59	6.27
2,4-*cis*-7-Hydroxyflavan-4-ol (**4**)	5.15	5.03	2.09	2.49	13.20	11.30	1.80	9.90	6.20
2,4-*trans*-5,7-Dihydroxyflavan-4-ol (**5**)	5.22	5.52	3.05	2.89	16.90	8.40	16.70	13.30	2.90

**Table 4:** UV absorption of 7-hydroxyflavanone **(1)** and its biotransformation products **(2 ÷ 6)**.

Compound	1st band		2nd band		3rd band	
	*λ*_max⁡_ [nm]	log *ε*	*λ*_max⁡_ [nm]	log *ε*	*λ*_max⁡_ [nm]	log *ε*
7-Hydroxyflavanone (**1**)	237	4.15	275	4.18	310	3.95
7-Methoxyflavanone (**2**)	235	4.18	273	4.19	310	3.89
3′,4′-Dihydroxy-7-methoxyflavanone (**3**)	238	4.25	272	4.39	335	4.01
2,4-*cis*-7-Hydroxyflavan-4-ol (**4**)	220	3.95	282	4.20	—	—
2,4-*trans*-5,7-Dihydroxyflavan-4-ol (**5**)	237	4.08	284	4.46	—	—
7-Hydroxyflavone (**6**)	254	4.32	302	4.34	—	—

**Table 5:** The IC_50_ values of the 7-hydroxyflavanone **(1)** and the biotransformation products.

Substrate	Product	IC_50_* (±SD) [*μ*M]
7-Hydroxyflavanone (**1**)		9.44 (±0.06)
	3′,4′-Dihydroxy-7-methoxyflavanone (**3**)	6.70 (±0.07)
	2,4-*trans*-5,7-Dihydroxyflavan-4-ol (**5**)	7.07 (±0.05)
	7-Hydroxyflavone (**6**)	8.80 (±0.05)
	2,4-*cis*-7-Hydroxyflavan-4-ol (**4**)	9.17 (±0.03)
	7-Methoxyflavanone (**2**)	9.50 (±0.07)

*Mean values of IC_50_ calculated as an average of at least three measurements.

## References

[1] Harborne JB, Baxter H (1999). *The Handbook of Natural Flavonoids*.

[2] Wiczkowski W, Piskula MK (2004). Food flavonoids. *Polish Journal of Food and Nutrition Sciences*.

[3] Winkel-Shirley B (2001). It takes a garden. How work on diverse plant species has contributed to an understanding of flavonoid metabolism. *Plant Physiology*.

[4] Di Carlo G, Mascolo N, Izzo AA, Capasso F (1999). Flavonoids: old and new aspects of a class of natural therapeutic drugs. *Life Sciences*.

[5] Havsteen BH (2002). The biochemistry and medical significance of the flavonoids. *Pharmacology and Therapeutics*.

[6] Middleton E, Kandaswami C, Theoharides TC (2000). The effects of plant flavonoids on mammalian cells: implications for inflammation, heart disease, and cancer. *Pharmacological Reviews*.

[7] Sampson L, Rimm E, Hollman PCH, De Vries JHM, Katan MB (2002). Flavonol and flavone intakes in US health professionals. *Journal of the American Dietetic Association*.

[8] Harborne JB (1988). *The Flavonoids*.

[9] Chun HK, Ohnishi Y, Shindo K, Misawa N, Furukawa K, Horinouchi S (2003). Biotransformation of flavone and flavanone by *Streptomyces lividans* cells carrying shuffled biphenyl dioxygenase genes. *Journal of Molecular Catalysis B*.

[10] Maatooq GT, Rosazza JPN (2005). Metabolism of daidzein by Nocardia species NRRL 5646 and Mortierella isabellina ATCC 38063. *Phytochemistry*.

[11] Seeger M, González M, Cámara B (2003). Biotransformation of natural and synthetic isoflavonoids by two recombinant microbial enzymes. *Applied and Environmental Microbiology*.

[12] Wang A, Zhang F, Huang L (2010). New progress in biocatalysis and biotransformation of flavonoids. *Journal of Medicinal Plant Research*.

[13] Das S, Rosazza JPN (2006). Microbial and enzymatic transformations of flavonoids. *Journal of Natural Products*.

[14] Sanchez-Gonzalez M, Rosazza JPN (2004). Microbial transformations of chalcones: hydroxylation, O-demethylation, and cyclization to flavanones. *Journal of Natural Products*.

[15] Ibrahim AR, Abul-Hajj YJ (1990). Microbiological transformation of chromone, chromanone, and ring a hydroxyflavones. *Journal of Natural Products*.

[16] Herath W, Mikell JR, Hale AL, Ferreira D, Khan IA (2006). Microbial metabolism. Part 6. Metabolites of 3- and 7-hydroxyflavones. *Chemical and Pharmaceutical Bulletin*.

[17] Herath W, Mikell JR, Hale AL, Ferreira D, Khan IA (2008). Microbial metabolism part 9.1 Structure and antioxidant significance of the metabolites of 5,7-dihydroxyflavone (chrysin), and 5- and 6-hydroxyflavones. *Chemical and Pharmaceutical Bulletin*.

[18] Hosny M, Dhar K, Rosazza JPN (2001). Hydroxylations and methylations of quercetin, fisetin, and catechin by Streptomyces griseus. *Journal of Natural Products*.

[19] Buisson D, Quintin J, Lewin G (2007). Biotransformation of polymethoxylated flavonoids: access to their 4′-O-demethylated metabolites. *Journal of Natural Products*.

[20] Kostrzewa-Susłow E, Dmochowska-Gładysz J, Białońska A, Ciunik Z, Rymowicz W (2006). Microbial transformations of flavanone and 6-hydroxyflavanone by Aspergillus niger strains. *Journal of Molecular Catalysis B*.

[21] Kostrzewa-Susłow E, Dmochowska-Gładysz J, Janeczko T (2010). Microbial transformation of selected flavanones as a method of increasing the antioxidant properties. *Zeitschrift fur Naturforschung C*.

[22] Arora A, Nair MG, Strasburg GM (1998). Antioxidant activities of isoflavones and their biological metabolites in a liposomal system. *Archives of Biochemistry and Biophysics*.

[23] Soczyńska-Kordala M, Bakowska A, Oszmiański J, Gabrielska J (2001). Metal ion-flavonoid associations in bilayer phospholipid membranes. *Cellular and Molecular Biology Letters*.

[24] Van Acker SABE, De Groot MJ, Van Berg DJD (1996). A quantum chemical explanation of the antioxidant activity of flavonoids. *Chemical Research in Toxicology*.

[25] Van Acker SABE, Van Den Berg DJ, Tromp MNJL (1996). Structural aspects of antioxidant activity of flavonoids. *Free Radical Biology and Medicine*.

